# C-852-04. Maintaining Normothermia in Burn Patients: Hypothermia Prevalence and Clinical Impact at ED-to-ICU Transfer

**DOI:** 10.1093/jbcr/irag033.064

**Published:** 2026-04-06

**Authors:** Sarah Wang, Anika Y Kim, Diego A Gonzalves, Artur Manasyan, Zachary J Collier, Sabrina Bawa, Shaheen Emami, Christian Hernandez, Claudia Nevarez, Ellen Maniago, Matthew Wiepking, T Justin Gillenwater

**Affiliations:** Keck School of Medicine, University of Southern California, Los Angeles, CA; Division of Plastic and Reconstructive Surgery, Keck School of Medicine, University of Southern California, Los Angeles, CA; Keck School of Medicine, University of Southern California, Los Angeles, CA; Los Angeles General Medical Center Regional Burn Center, Los Angeles, CA; Division of Plastic and Reconstructive Surgery, Keck School of Medicine, University of Southern California, Los Angeles, CA; Department of Emergency Medicine, Department of Anesthesia, Keck School of Medicine, University of Southern California, Los Angeles, CA; Department of Emergency Medicine, Keck School of Medicine, University of Southern California, Los Angeles, CA; Department of Emergency Medicine, Keck School of Medicine, University of Southern California, Los Angeles, CA; Los Angeles General Medical Center Regional Burn Center, Los Angeles, CA; Los Angeles General Medical Center Regional Burn Center, Los Angeles, CA; Department of Emergency Medicine, Keck School of Medicine, University of Southern California, Los Angeles, CA; Division of Plastic and Reconstructive Surgery, Keck School of Medicine, University of Southern California, Los Angeles, CA

## Abstract

**Introduction:**

Hypothermia is a common complication in burn patients during the acute post-burn phase due to loss of thermoregulation from a compromised skin barrier. Hypothermia at the time of admission to the burn intensive care unit (ICU) is associated with a 5% increase in mortality for every 1.0°C drop in body temperature below 36.0°C. The prevalence of hypothermia at emergency department (ED)-to-ICU handoff remains substantial, with rates ranging from 20% to 60%. Given the significant impact on burn mortality, this study examines the prevalence and effects of hypothermia in the ED of an urban trauma center prior to burn ICU transfer.

**Methods:**

A retrospective review of burn patients who were admitted to a Level I trauma center burn ICU from 2022 to 2025 was performed. Initial, lowest, and final temperatures during patients’ ED stays were recorded. Hypothermia was defined as a temperature lower than 36.0°C. Tracked outcomes included demographics, total body surface area (TBSA), inhalation injury, length of stay (LOS), ICU LOS, and mortality. Descriptive statistics and multivariate analysis were used with a significance level set at *p*-value <0.05.

**Results:**

A total of 188 burn ICU patients were identified and 11 were then excluded for incomplete ED temperature records. For the remaining 177 burn ICU patients, the average age was 44.4 years old, and 132 (74.6%) patients were male. The average TBSA was 22.9% (95% CI = 19.4 - 26.3), ICU LOS was 9.0 days (95% CI = 6.7 - 11.2), total LOS was 20.1 days (95% CI = 15.7 - 24.6), and 52 (29.4%) patients experienced inhalation injury. Mean ED temperatures were: initial 36.3°C (95% CI = 36.1 - 36.4), lowest 35.8°C (95% CI = 35.6 - 36.0), and final 36.2°C (95% CI = 36.0 - 36.4). Hypothermia was present in 54 (30.5%) patients. In 69 (39.0%) patients, the final ED temperature at the time of transfer to burn ICU was lower than the initial admission temperature. The overall mortality rate was 18.6% (33/177), increasing to 31.5% (17/54) among patients with hypothermia. After adjusting for confounders, there was no significant association between hypothermia and total or ICU LOS. Among hypothermic patients, lower ED temperature at time of transfer was significantly associated with higher mortality (*p*=.02). For every 1°C decrease below 36°C at time of transfer, there was a doubling of the odds of death at discharge (95% CI = 1.1 – 3.6).

**Conclusions:**

Hypothermia occurred in nearly one third of burn patients in the ED prior to burn ICU transfer. Greater severity of hypothermia at transfer was independently associated with increasing mortality. These findings support implementation of early, protocolized warming and continuous temperature monitoring as actionable targets during the ED phase to mitigate adverse effects of hypothermia.

**Applicability of Research to Practice:**

Future interventions for burn patients in the ED should be aimed at maintaining normothermia through an interdisciplinary approach to reduce mortality.

**Funding for the study:**

N/A.

